# Acinetobacter baumannii: trends in antimicrobial resistance after relocation of an intensive care unit in tunisia

**DOI:** 10.1186/2197-425X-3-S1-A135

**Published:** 2015-10-01

**Authors:** S Koubaji, S Kamoun, A Ben Souissi, F Haddad, Y Ben Aicha, MS Mebazaa

**Affiliations:** Anesthesiology and ICU Department, Mongi Slim University Hospital La Marsa, Sidi Daoued, Tunisia

## Purpose

Multidrug-resistant Acinetobacter Baumannii (MDRAB) is a worldwide threat because of its remarkable ability to survive in harsh environment for months. It has been shown that MDRAB can colonize patient environment [1], especially in intensive care units (ICU). This study aims to compare the prevalence and antimicrobial susceptibility pattern of Acinetobacter Baumannii infecting ICU patients before and after relocation.

## Methods

A retrospective study including all patients with Acinetobacter Baumannii (AB) infections, admitted in ICU between January 2013 and January 2015; the relocation occurred in September 2013. We recorded the site of infection, the antimicrobial susceptibility, antibiotic therapy and the outcome.

## Results

310 patients were hospitalized in intensive care unit: 160 in the old location and 150 in the new one. Among the 310 patients, 36 developed infection with AB isolated in 45 cultures: 17 in the old location and 19 in the new one. In the old location, isolates were resistant to imipenem in 66%, 100% to amikacin, 100% to quinolones, and 16% susceptible to fosfomycin, 94.11% susceptible to colistin, 94% to rifampicin and 100% to tigecycline, however in the new location, we noted 95% resistance to imipenem, 21% resistance to amikacin, 100% resistance to quinolones, and 20% susceptibility to fosfomycin, 100% to colistin, 94.7% to rifampicin and 100% susceptible to tigecycline. All patients have received a combination therapy adapted to microbiological findings: 2.7% Fosfomycin + colistin, 13.8% imipenem + colistin, 38.8% colistin + Rifampicin, 44.7% Tigecycline + colistin. The mortality rate was 72.3%: 74% related to refractory septic shock and 26% due to different causes.

## Discussion

The main risk factors for nosocomial infections in ICUs are: assisted ventilation, large prescription of antibiotics, prolonged hospital stay and catheterization. Stringent measures had to be applied: restricted policy for the use of specific drugs, septic isolation, disinfection of the environment and even the closure of the unit. Furthermore, the resistance of the AB affects different classes of antibiotics such as carbapenems. However, resistance to imipenem is variable according to the authors, ranging from 3.1 to 60% [2]. No resistance of AB to colistin was found according to various studies [3]. For multidrug-resistant AB, the antibiotherapy with colistin, rifampicin and tigecycline [4] have been associated with” favorable” clinical outcomes.

## Conclusions

The spread of multidrug-resistant AB in ICU presents a challenge to clinician. It is necessary to intensify efforts to respect hygiene rules and restriction in the use of antibiotics.
